# Naturally selected CD7-directed CAR-T bridging allo-HSCT in refractory acute myeloid leukemia: a case report and review

**DOI:** 10.3389/fimmu.2024.1461908

**Published:** 2024-10-14

**Authors:** Xuan Liu, Zheng Xu, Shuhui Li, Xuejun Zhang, Jianqiang Li, Hang Li, Fuxu Wang

**Affiliations:** ^1^ Department of Hematology, The Second Hospital of Hebei Medical University, Hebei Key Laboratory of Hematology, Shijiazhuang, Hebei, China; ^2^ Hebei Senlang Biotechnology Co, Shijiazhuang, China; ^3^ Department of Histology and Embryology, Hebei Medical University, Shijiazhuang, Hebei, China

**Keywords:** relapsed/refractory, acute myeloid leukemia, naturally selected CD7 CAR-T, HSCT, CRS

## Abstract

Relapsed/refractory acute myeloid leukemia (R/R-AML) has a poor prognosis. CD7 is expressed in leukemic cells in 30% of patients with AML but not in normal myeloid cells. Therefore, it can be a potential target for immunotherapy in patients with R/R-AML. Naturally selected CD7-directed chimeric antigen receptor T cells (CAR-T) have promising effects against AML based on xenotransplantation models. We report a R/R-AML case that achieved complete remission with incomplete hematologic recovery with naturally selected CD7 CAR-T therapy. Allogeneic hematopoietic stem cell transplantation (allo-HSCT) as consolidation early after CAR T therapy, the patient experienced 12 months of disease-free survival to date. Our results confirmed that allogeneic hematopoietic stem cell transplantation after naturally selected CD7 CAR-T therapy can be a potential treatment for patients with CD7-positive R/R-AML.

## Background

Patients with relapse/refractory (R/R-AML) have an overall poor prognosis. The long-term survival rate of patients with R/R-AML is approximately 10%, and the median survival time is approximately 1 year (1). Novel drugs such as BCL-2, IDH1/IDH2, and FLT3 inhibitors, have improved the prognosis of some AML patients. However, owing to the limitations of novel targeted drugs, patients with R/R-AML without a target are recommended for clinical trials. At present, several tumor antigens, such as CD33, CD123, CD7, C-type lectin-like molecule 1, CD38, TIM3, CD70, FLT3, and CD47, have been explored as target antigens for AML therapy (2–6). CD7 is expressed in T/NK and its progenitor cells only, not in normal myeloid hematopoietic cells. Naturally selected CD7 CAR-T cell therapy has tumor-killing effects based on in vitro and AML xenotransplantation models, whereas, it has no cytotoxic effects on normal myeloid cells (7). Herein, we report a case of CD7+R/R-AML treated with allogeneic hematopoietic stem cell transplantation after naturally selected CD7 CAR-T therapy.

## Case presentation

A 48-year-old female patient was admitted to the Department of Hematology, The Second Hospital of Hebei Medical University (Shijiazhuang, China) in February 2022 for 20 days of ecchymosis. Hematological analysis revealed a white blood cell count of 293×10^9^/L, a hemoglobin count of 72 g/L, and a platelet count of 12×10^9^/L. Blasts in bone marrow were 80.5%. Flow cytometry (FCM) revealed that approximately 78.28% of abnormal cells expressed HLA-DR, CD34, CD123, CD38, CD117, CD7, CD64, CD11c, CD13, and CD33. Chromosome karyotyping revealed a normal karyotype 46,XX. The acute leukemia fusion gene screening was negative. Next-generation sequencing revealed FLT3-ITD, KIT, NRAS, and CEBPA mutations. Pulmonary tuberculosis was observed at the initial diagnosis and was treated with triple antituberculosis therapy for half a year.

**Figure 1:**
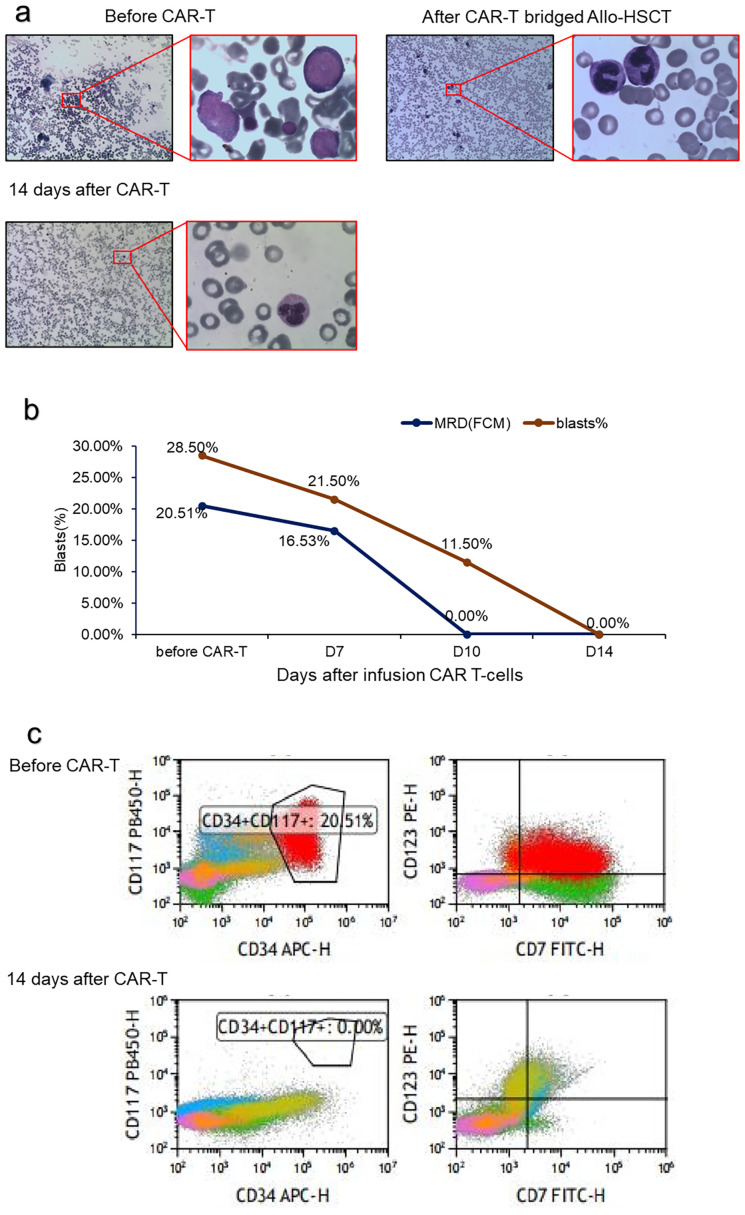
Treatment response of CD7 CAR T-cell infusion. **(A)** BM morphology before and after CD7 CAR T-cell infusion; **(B)** Change in the percentage of blasts and MRD in BM after CD7 CAR T-cell infusion; **(C)** Flow cytometry analysis in BM before and after CD7 CAR T-cell infusion.

Complete remission (CR) was achieved with induction chemotherapy with venetoclax, Decitabine, and sorafenib. Recurrence occurred more than 1 month after consolidation treatment with the same regimen. Furthermore, multiple subcutaneous nodules developed simultaneously. A skin biopsy revealed that the dermis presented with multifocal myeloblastic cell infiltration, combined with an immunophenotype as follows: CD117 (focal +), CD138 (−), CD20 (−), CD3 (−), CD30 (−), CD34 (focal +), CD38 (−), CD5 (−), CD68 (+), CD99 (+), Ki67 (+), MPO (+), and PAX-5 (−). Bone marrow cytology showed that the myeloblast percentage was 21%. Next-generation sequencing revealed CEBPA mutations but not KIT-D816V and FLT3-ITD mutations. A mitoxantrone liposome plus cytarabine regimen was administered to achieve CR2 and was consolidated with the same regimen. MRD was still positive. She was refused allo-HSCT in another hospital for active TB and was treated with a daratumumab, avapritinib, and selinexor regimen. AML relapsed again 3 weeks later. After returning to our hospital, she was retreated with the mitoxantrone liposome and cytarabine regimen but had no response. Blast cells were 28.5% in the bone marrow. Flow cytometry revealed 20.5% of the bone marrow nucleated cells were leukemic cells. Almost all leukemic cells expressed CD7 (**Figure 1**). The patient had relapsed and refractory acute myeloid leukemia.

Hence, the patient received compassionate therapy with naturally selected CD7 CAR-T(NS7CAR). The CAR-T cells were engineered by Hebei Senlangbio Technology Co., Ltd. (Shijiazhuang, China). Peripheral blood mononuclear cells (PBMCs) from patients were isolated by density gradient centrifugation. T cells were purified using CD3 magnetic beads. The CAR-T cells were obtained by transducing total T cells and then culturing them using the natural selection cell culture method. NS7CAR escape the self-destructive cell killing mechanism due to the antigen-masking/intracellular sequestration effect. The design and production of naturally selected CD7 CAR-T cells was performed as described previously (7, 8). Before CAR-T reinfusion, lymphodepleting chemotherapy with the FC regimen (30 mg/m^2^ of fludarabine, day -5 to -3; 300 mg/m^2^ of cyclophosphamide, day -5 to -3) was administered. The number of CAR-T cells was 5 × 10^5^/kg. The expansion of CAR cells and the level of cytokines were monitored 4, 7, 10, and 14 days after transfusion.

**Figure 2:**
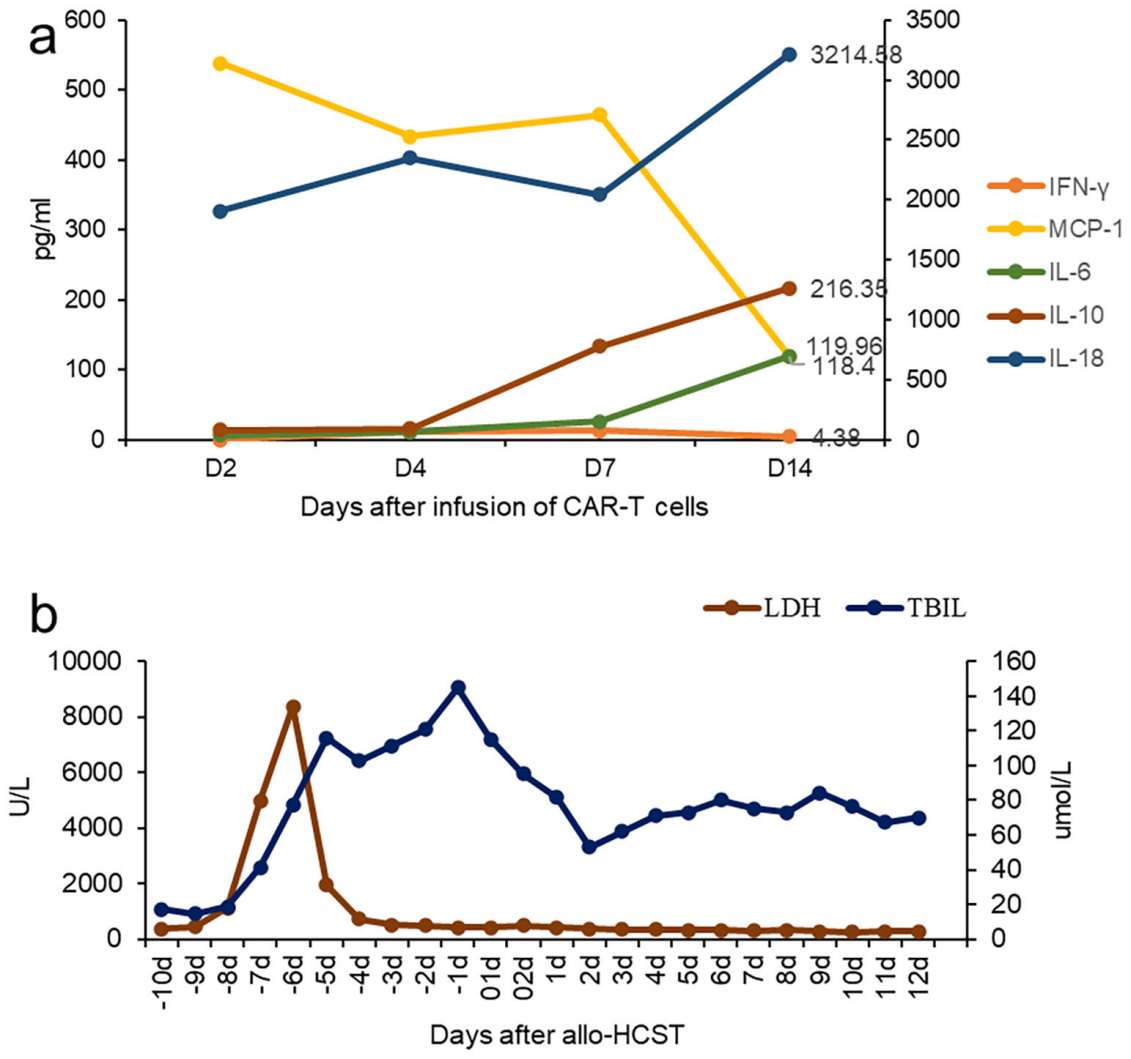
Clinical characteristics after CAR T-cell infusion. **(A)** Change in cytokines after CD7 CAR T-cell infusion. **(B)** The changes in bilirubin and lactate dehydrogenase during the Allo-HCST process.

After 1 week, the number of CAR cells increased (**Table 1**). After 2 weeks, the bone marrow blast cells were <5%, and MRD negativity (FCM) was achieved (**Figure 1**). Patients were assessed for complete remission with incomplete hematologic recovery. Transient fever occurred after cell infusion, and the highest body temperature was greater than 38°C. However, hypotension, hypoxemia, and adverse reactions of the nervous system were not observed. Cytokines IL-6, IL-10, and IL-18 increased significantly after cell infusion, but the cytokine IFN-γ level was not increased (**Figure 2A**). The patient was diagnosed with grade 1 cytokine release syndrome. The patient’s body temperature was controlled after the short-term use of dexamethasone (9).

**Table 1:** CAR DNA copies per microgram of genome.

Acquisition time	CAR-7	
	Peripheral blood	Bone marrow
4 days after CAR-T	7.56 × 10^1^	
7 days after CAR-T	1.15 × 10^3^	5.27 × 10^2^
10 days after CAR-T		7.29 × 10^4^
14 days after CAR-T	1.5 × 10^5^	2.37 × 10^5^
17 days after CAR-T		8.32 × 10^4^
42 days after CAR-T	1	
59 days after CAR-T	1	

The patient has a human-leukocyte-antigen (HLA)-haploid-matched daughter, who was willing to be a donor for the hematopoietic stem cell transplantation (HSCT). On 22 November 2022, she underwent allogeneic transplantation with peripheral blood hematopoietic stem cells from her daughter 3 weeks after CAR-T therapy. The preconditioning regimen included busulfan (3.2 mg/kg/day) on day −6, fludarabine (30 mg/m^2^) on days −5 to −2, cyclophosphamide (1.8 g/m^2^/day) on day −3 and −2, and rabbit anti-human thymocyte globulin (ATG, 2.5 mg/kg/day, SangStat, Lyon, France) on days −5 to −2. The graft-versus-host disease (GVHD) prophylaxis was based on cyclosporine, mycophenolate mofetil (MMF), and a short course of methotrexate. Donor mononuclear cells of 10.7×10^8^/kg, including CD34-positive cells of 4.07×10^6^/kg, were transfused. Neutrophil and platelet engraftments were observed on day +20, and a full-donor-chimerism of 97.5% was evidenced on day +28. Furthermore, the patient received acyclovir and letermovir, which can prevent herpesvirus and cytomegalovirus infection, respectively. Liver function injury occurred during preconditioning, which improved after glucocorticoid and symptomatic treatment (**Figure 2B**). On day +10, the diagnosis of early thrombotic microangiopathy (TMA) was made based on hypertension, urinary protein (2+), serum creatinine and LDH levels, and serous effusion (pleural and pericardial effusion). Moreover, sC5b-9:835.3 ng/ml were noted. After the discontinuation of cyclosporine and substitution with CD25 monoclonal antibody, and the addition of defibrotide for TMA, the patient’s condition improved. During follow-up at 12 months after transplantation, the primary disease was in CR, and the patient did not present with acute graft-versus-host disease.

## Discussion

Approximately 30% of patients with AML express CD7, and CEBPA mutations may be associated with a high CD7 expression (7, 10–13). CD7 expression is a poor prognostic factor (14, 15). Therefore, CD7 CAR-T cells can be considered for patients with CD7+ R/R-AML. However, CD7 chimeric antigen receptor (CAR) T cells undergo fratricide that impairs the effect of CAR T cells. The researchers used gene editing (16, 17), protein blocker (18), recombinant anti CD7 blocking antibodies (19), natural selection CD7 CAR T-cells (7) and other methods to obtain CD7 CAR-T cells, which improved the success rate of CAR-T cell preparation and maintained their activity. To date, two cases of AML treated with CD7 CAR-T cells have been reported (20, 21)(**Table 2**), and clinical trials are under way (**Table 3**). Naturally selected CD7 CAR-T cells have shown a good safety profile in the treatment of T-ALL/LBL (8).

**Table 2:** Clinical cases of AML treated with CD7 CAR T cells.

	Number of cases	CD7+ in blast cell	Blast cell in BM	EMD	Source of CAR T cells	CAR T dose (10^7^/kg)	CRS	ICANS	Clinical outcome
Yongxian Hu et al.	1	75%	51%	NO	Healthy donors	2	1	None	CRi
Xuanqi Cao et al.	1	95.6%	20%	NO	autologous	0.5	3	None	CR

**Table 3:** Summary of active and completed clinical trials according to www.clinicaltrials.gov.

NCT Number	Study title	Study status	Interventions	Locations
NCT04599556	Clinical Trial for the Safety and Efficacy of Anti-CD7 CAR-T Cell Therapy for Patients With Relapsed or Refractory CD7 Positive Hematological Malignancy	RECRUITING	BIOLOGICAL: anti-CD7 CAR-T	The First Affiliated Hospital College of Medicine, Zhejiang University, Hangzhou, Zhejiang, 310003, China
NCT04762485	Humanized CD7 CAR T-cell Therapy for r/r CD7+ Acute Leukemia	UNKNOWN	BIOLOGICAL: Humanized CD7 CAR-T cells	The First Affiliated Hospital of Soochow University, Suzhou, (Select), 215000, China
NCT05454241	CD7 CAR-T for Patients With r/r CD7+ Hematologic Malignancies	RECRUITING	DRUG: Anti-CD7 CAR-T	Institute of Hematology & Blood Diseases Hospital, Tianjin, 300020, China
NCT05827835	CD7 CAR-T Bridging to alloHSCT for R/R CD7+Malignant Hematologic Diseases	RECRUITING	DRUG: CD7 CAR-T cells injection|OTHER: Allogeneic hematopoietic stem cell transplantation	The First Affiliated Hospital of Medical College of Zhejiang University, Hangzhou, Zhejiang, 310003, China
NCT04033302	Multi-CAR T Cell Therapy Targeting CD7-positive Malignancies	UNKNOWN	BIOLOGICAL: CD7-specific CAR gene-engineered T cells	Shenzhen Geno-immune Medical Institute, Shenzhen, Guangdong, 518000, China
NCT05995028	Universal 4SCAR7U Targeting CD7-positive Malignancies	RECRUITING	BIOLOGICAL: Universal CD7-specific CAR gene-engineered T cells	Shenzhen Geno-immune Medical Institute, Shenzhen, Guangdong, 518000, China
NCT05377827	Dose-Escalation and Dose-Expansion Study to Evaluate the Safety and Tolerability of Anti-CD7 Allogeneic CAR T-Cells (WU-CART-007) in Patients With CD7+ Hematologic Malignancies	RECRUITING	BIOLOGICAL: WU-CART-007	Washington University School of Medicine, Saint Louis, Missouri, 63110, United States

In the current case, the patient presented with a CEBPA mutation and high CD7 expression. The leukemia in the patient was relapsed and refractory and accompanied by extramedullary lesions. Considering the high risk of recurrence after transplantation, the patient received compassionate treatment with naturally selected CD7 CAR-T transfusion. Because we were concerned about long-term myelosuppression, which could increase the risk of infection and bleeding, hematopoietic stem cell transplantation was performed as soon as possible to promote hematopoietic recovery. During the follow-up of 12 months after transplantation, the patient continued to achieve complete remission of the primary disease, and no acute graft-versus-host disease occurred. The result suggests that naturally selected CD7 CAR T-cell therapy is an encouraging approach to the treatment of CD7-positive R/R AML. However, early thrombotic microangiopathy after transplantation was observed during the treatment course, and some studies have shown that the inflammatory reaction of CAR-T after treatment may lead to endothelial injury (22). Whether undergoing allogeneic hematopoietic stem cell transplantation early after CAR T-cell therapy increases the risk of TMA remains unclear. Currently, the number of cases is small, and the remission time of CAR-T after treatment and the timing of transplantation should be evaluated clinically.

## Data Availability

The original contributions presented in the study are included in the article/supplementary material. Further inquiries can be directed to the corresponding author.
